# Sex- and Age-Related Estrogen Signaling Alteration in Inflammatory Bowel Diseases: Modulatory Role of Estrogen Receptors

**DOI:** 10.3390/ijms20133175

**Published:** 2019-06-28

**Authors:** Damian Jacenik, Adam I. Cygankiewicz, Anna Mokrowiecka, Ewa Małecka-Panas, Jakub Fichna, Wanda M. Krajewska

**Affiliations:** 1 Department of Cytobiochemistry, Faculty of Biology and Environmental Protection, University of Lodz, Pomorska St. 141/143, 90-236 Lodz, Poland; 2 Department of Digestive Tract Diseases, Faculty of Medicine, Medical University of Lodz, Stefana Kopcinskiego St. 22, 90-001 Lodz, Poland; 3 Department of Biochemistry, Faculty of Medicine, Medical University of Lodz, Mazowiecka St. 6/8, 92-215 Lodz, Poland

**Keywords:** inflammatory bowel diseases, Crohn’s disease, ulcerative colitis, estrogen receptors, GPER, ERα, ERβ, ERα36, ERα46

## Abstract

The pathogenesis of inflammatory bowel diseases (IBD) seems to be associated with alterations of immunoregulation. Several lines of evidence suggest that estrogens play a role in the modulation of immune responses and may be related to the etiology of IBD. The purpose of this work was to examine the involvement of G protein-coupled estrogen receptor (GPER), estrogen receptor α (ERα), estrogen receptor β (ERβ) and ERα spliced variants ERα36 and ERα46 in Crohn’s disease (CD) and ulcerative colitis (UC). The studied group included 73 patients with IBD and 31 sex and age-related controls. No differences in serum levels of 17β-estradiol nor of CYP1A1 and SULT1E1 enzymes involved in estrogen catabolism were stated. The expression pattern of estrogen receptors in tissue samples was quantified using real-time PCR and Western blotting. Statistically significant up-regulation of GPER and ERα in both CD and UC as well as down-regulation of ERβ in CD patients was found. However, differences in the expression of estrogen receptors in CD and UC have been identified, depending on the sex and age of patients. In men, up-regulation of GPER, ERα and ERα46 expression was shown in CD and UC patients. In women under 50 years of age, GPER protein level increased in UC whereas ERβ expression tended to decrease in CD and UC patients. In turn, in women over 50 the protein level of ERα increased in UC while ERβ expression decreased in CD patients. Dysregulation of estrogen receptors in the intestinal mucosa of patients with CD and UC indicates that estrogen signaling may play a role in the local immune response and maintain epithelial homeostasis in a gender- and age-dependent manner.

## 1. Introduction

Crohn’s disease (CD) and ulcerative colitis (UC), the most commonly diagnosed types of inflammatory bowel diseases (IBD), are complex, immunologically mediated diseases of the gastrointestinal tract. Despite the differences between CD and UC pathology several phenomena such as dysregulation of the immune response, the gut microbiome, as well as genetic and environmental factors seem to be crucial in IBD pathogenesis [1,2,3]. Males are more likely to develop UC, whereas females are more likely to develop CD [4,5]. However, the molecular mechanisms for this bias remains unclear.

Estrogens play a complex role in the pathophysiology and accumulated data suggest the impact of estrogens on IBD [6,7,8,9,10]. There is evidence showing a higher prevalence of both IBD types with a relative risk of 1.65 for CD and 1.35 for UC in women [11]. A number of epidemiological studies have shown that exogenous hormone supplementation such as oral contraceptives (OC) or hormone replacement therapy (HRT) in postmenopausal women is associated with the occurrence of IBD. A meta-analysis by Ortizo et al. [12] indicated that OC users are characterized by a 24% and 30% higher risk for developing CD and UC, respectively. The positive relationship between OC use and risk of developing CD was noted in a large prospective cohort study [6]. Additionally, OC administration, and particularly the combination type, in long-term users was related with a higher possibility for CD-related surgery [7]. An association between HRT and increased risk of UC was also documented. The risk of UC appeared to increase with longer duration of hormone use. The contribution of HRT to CD is controversial. It has been suggested that HRT has no effect on Crohn’s disease [8]. On the other hand, in case-control analysis a positive relationship between HRT and CD development was found [9]. In contrast, the anti-inflammatory effects of estrogen supplementation in IBD has been demonstrated. Kane et al. [10] documented a dose-dependent protective property of HRT on disease severity in IBD.

Estrogen activity is the result of interaction with the relevant receptors and triggering estrogen-dependent effects on signaling pathways. In the classic pathway, ligand binds to the nuclear estrogen receptors (ERα and ERβ), which leads to changes in receptor conformation, dissociation of heat shock proteins (HSPs) and homo- or hetero-dimerization. Dimers are translocated to the cell nucleus, where by direct interaction with specific DNA sequences or indirectly *via* transcription factors, they regulate the expression of target genes on the genomic/long-term pathway. In addition to the classical genomic effects, the short-term/non-genomic action of estrogens is mediated by membrane-associated G protein-coupled estrogen receptor (GPER, formerly GPR30). Ligand-dependent activation of GPER leads to rapid activation of several proteins involved in signal transduction resulting in changes at the transcriptional level [13]. It is noteworthy, that there are also alternatively spliced variants of wild type ERα, i.e., ERα36 and ERα46, which are able to mediate both the non-genomic and genomic action of estrogens [13,14]. A growing body of evidence shows that estrogen signaling is a complex network, that is regulated by cross-talk between estrogen receptors, dependent on the tissue expression pattern of estrogen receptors [13,15].

The growing body of literature data emphasize the relationship between estrogen signaling and inflammation [16,17,18]. Based on epidemiological and experimental data, it is suggested that estrogen receptors α and β are important mediators of intestine inflammation. Although ERβ plays a minor role in mediating action in classical estrogen target tissues, it appears that this receptor plays an important role in the colon. Notably, ERβ expression was decreased in the colonic mucosa of IBD patients with active disease [19,20]. However, it has been proven that both nuclear estrogen receptors modulate epithelial barrier function. The findings of Goodman et al. [21] suggest that differences in ERα/ERβ signaling ratio affect colitis in males and females. In addition, our recent studies have shown that GPER expression is associated with colon inflammation in murine model of CD, and ligands acting through the appropriate estrogen receptors affect colitis not only at the macroscopic and microscopic levels, but also appear to be important regulators of signal transmission and immunomodulatory genes expression in the colon [22].

In our study, we investigated the involvement, not only of nuclear estrogen receptors ERα and ERβ, but also G protein-coupled estrogen receptor (GPER) and truncated forms of ERα, i.e., ERα36 and ERα46, in Crohn’s disease and ulcerative colitis and their relationship with gender and age. Better understanding of the role of estrogens receptors may advance our knowledge and the therapeutics or prevention strategies for IBD.

## 2. Results

### 2.1. Patients Characteristics

A total of one hundred and four patients hospitalized from 2011 to 2017 in the Department of Digestive Tract Disease, Faculty of Medicine at the Medical University of Lodz, Poland were enrolled for this study. Serum and forceps endoscopic biopsies of colon were collected from 31 CD patients (19 men, seven women under 50 and five women over 50 years old), 42 UC patients (24 men, 12 women under 50 and six women over 50 years old) and 31 unrelated controls (12 men, nine women under 50 and ten women over 50 years old). All blood parameters such as white blood cells, neutrophils, lymphocytes, monocytes, eosinophils, basophils, red blood cell counts and hemoglobin, sodium and potassium levels were in the normal range. However, the level of C-reactive protein (CRP), which is a biomarker of inflammation, was more than 30-fold and 33-fold higher in patients with CD and UC, respectively, compared to the control group (Table 1). In IBD patients in relation to the control group, a statistically significant increase of mRNA level for interleukin-6 (IL-6) that acts as pro-inflammatory cytokine was also observed (Table 2). As shown in Table 2, the statistically significant up-regulation at the mRNA level of one of the most highly expressed cytokines in human chronic inflammatory diseases that is involved in immune cell trafficking, i.e., CCL18 was stated in UC but not in CD patients compared to controls. Similarly, higher, but not statistically significant interleukin-10 (IL-10) transcript level in patients with UC but not with CD compared to the control group was noted. Our analysis demonstrated no changes in tumor necrosis factor-α (TNF-α) and nuclear factor kappa-light-chain-enhancer of activated B cells (NFκB) mRNA level in colon samples obtained from IBD patients (Table 2). There were no differences in the parameters tested in relation to the sex and age of patients.

### 2.2. 17β-Estradiol, CYP1A1 and SULT1E1 Levels in Serum of IBD Patients

**Figure 1:**
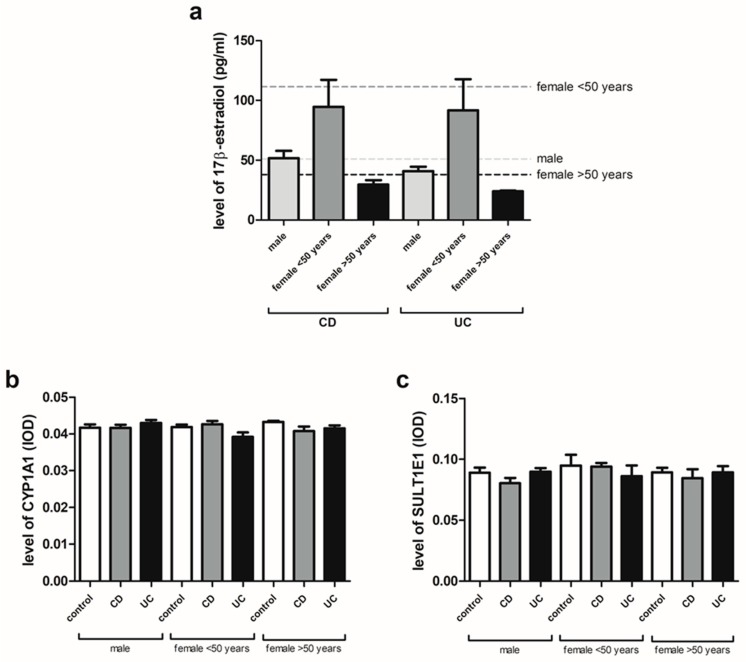
Serum 17β-estradiol (**a**), CYP1A1 (**b**) and SULT1E1 (**c**) levels in CD and UC patients and related controls: males (control, *n* = 12; CD, *n* = 19; UC, *n* = 24), females under the age of 50 years (control, *n* = 9; CD, *n* = 7; UC, *n* = 12) and females over the age of 50 years (control, *n* = 10; CD, *n* = 5; UC, *n* = 6). Dotted lines represent reference values. Values are means ± SEM.

The serum level of 17β-estradiol in males and females under and over the age of 50 years with IBD was found to be in the normal range. In the case of CD, the values were 51.74 ± 6.24 for men, 94.73 ± 22.58 for women under 50 years and 29.50 ± 3.75 for women over 50 years and in the case of UC 40.84 ± 3.69 for men, 91.63 ± 26.15 for women under 50 years and 23.89 ± 0.73 for women over 50 years (Figure 1a). As shown in Figure 1b, no differences were observed in the serum level of CYP1A1 (cytochrome P450 family 1 subfamily A member 1) for men and women under and over the age of 50 years with CD and UC compared to the control group. Similarly, no changes in the SULT1E1 (sulfotransferase family 1E member 1) serum level were found in the studied IBD forms in both men and women regardless of age (Figure 1c).

### 2.3. Estrogen Receptors Expression in IBD Patients

**Figure 2:**
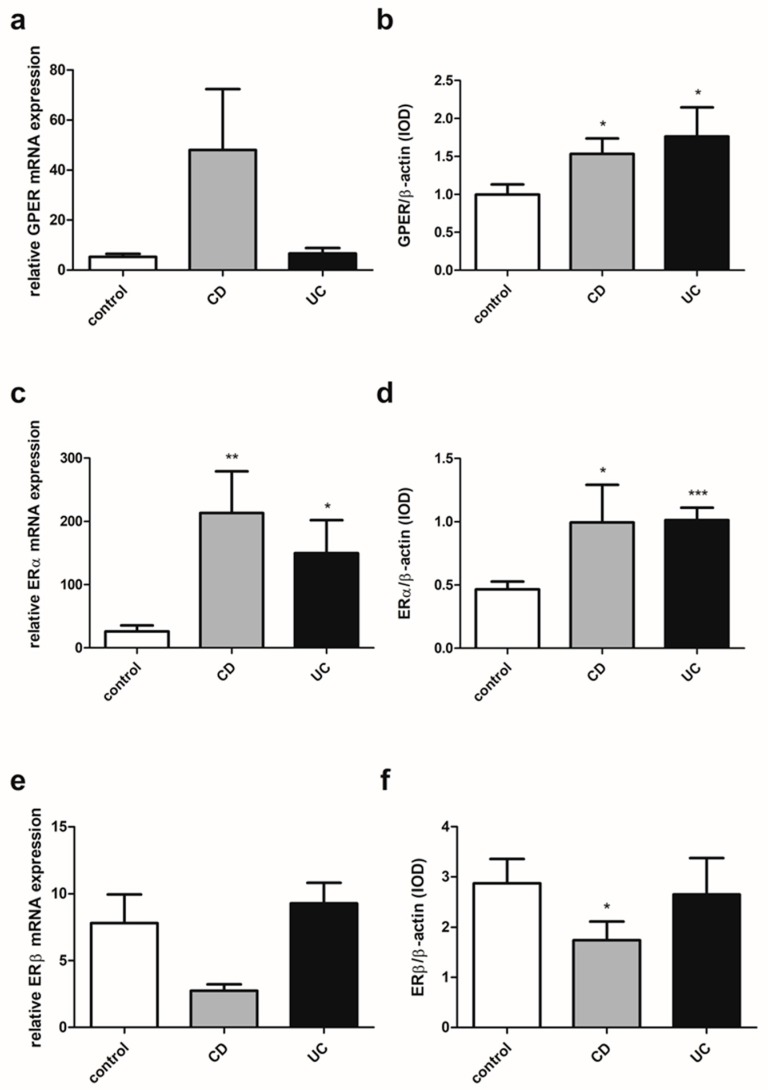
Expression of GPER (**a**,**b**), ERα (**c**,**d**) and ERβ (**e**,**f**) at the mRNA (**a**,**c**,**e**) and protein level (**b**,**d**,**f**) in CD and UC patients and the control group regardless of sex and age (control, *n* = 31; CD, *n* = 31; UC, *n* = 42). Values are means ± SEM; * *P* < 0.05, ** *P* < 0.01, *** *P* < 0.001 vs. control.

In colon samples obtained from patients with IBD, regardless of gender and age, despite the lack of statistically significant differences in GPER expression at the mRNA level up-regulation of the GPER protein level was demonstrated in both CD (*P* = 0.012) and UC (*P* = 0.019) in relation to the control group (Figure 2a,b). For ERα, a statistically significant increase in expression was revealed both at the level of mRNA and protein in CD (*P* = 0.004 and *P* = 0.028, respectively) and UC patients (*P* = 0.021 and *P* < 0.0001, respectively) compared to the control group (Figure 2c,d). In the case of ERβ expression analysis, a lower mRNA level and statistically significant (*P* = 0.043) decrease of ERβ protein level were observed in patients with CD (Figure 2e,f). No differences were found in ERβ mRNA and protein expression in patients with UC (*P* = 0.167 and *P* = 0.441, respectively) in relation to the control group.

**Figure 3:**
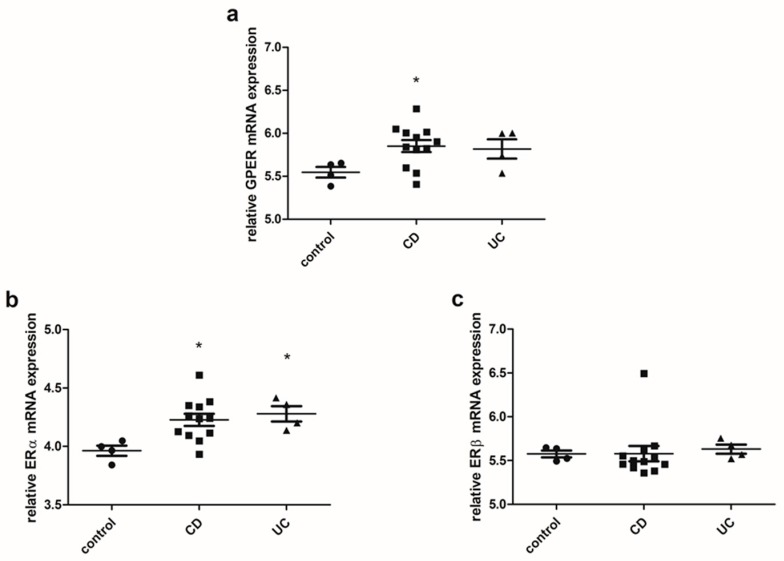
Expression of GPER (**a**), ERα (**b**) and ERβ (**c**) at the mRNA level in CD and UC patients and the control group regardless of sex and age (control, *n* = 4; CD, *n* = 12; UC, *n* = 4). Values are means ± SEM; * *P* < 0.05 vs. control.

Estrogen receptor analysis was also performed in an independent cohort using the dataset provided by Global Expression Omnibus (GEO; accession number: GSE6731). Colon samples from CD patients were characterized by statistically significant up-regulation of GPER (*P* = 0.045) at the mRNA level corresponding to the control group (Figure 3a). As shown in Figure 3b, in both CD (*P* = 0.018) and UC (*P* = 0.028) patients higher statistically significant mRNA level of ERα was revealed. No statistically significant differences in the case of ERβ mRNA analysis were observed in colon samples from IBD patients in comparison to the control group (Figure 3c).

### 2.4. Estrogen Receptors Expression in Men with IBD

**Figure 4:**
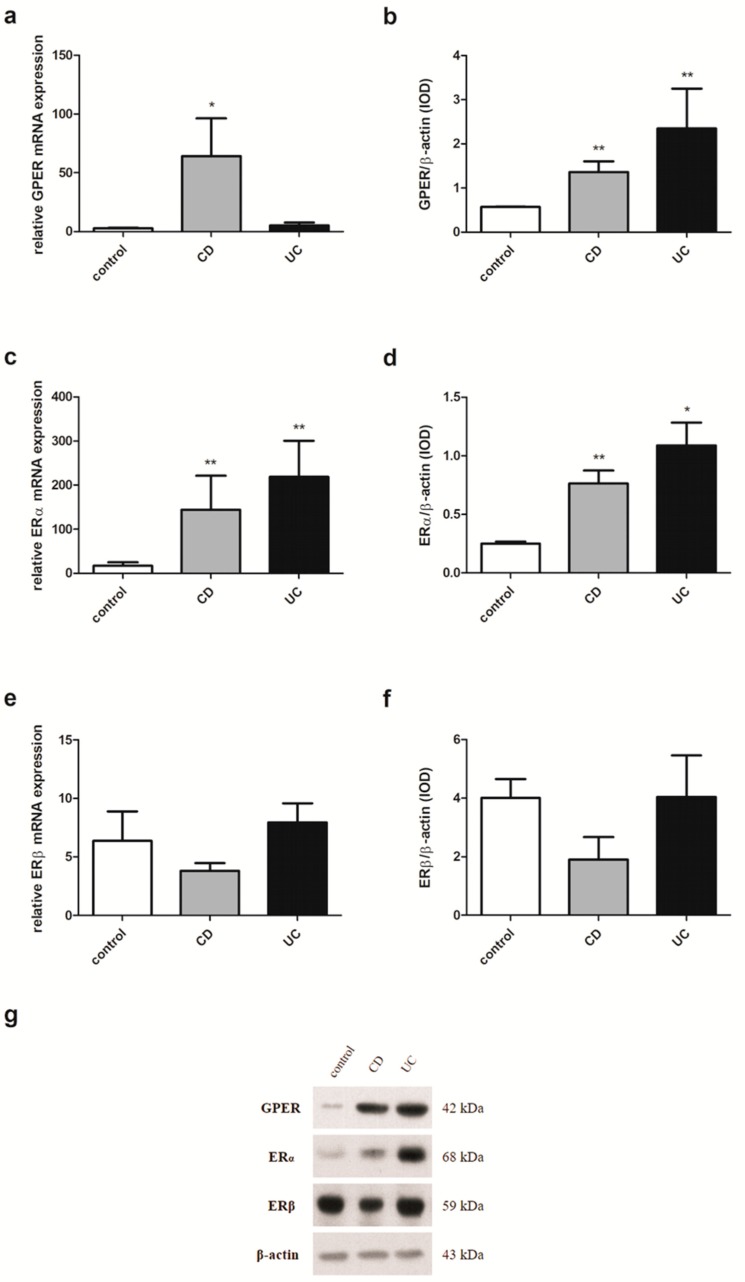
Expression of GPER (**a**,**b**), ERα (**c**,**d**) and ERβ (**e**,**f**) at the mRNA (**a**,**c**,**e**) and protein level (**b**,**d**,**f**) in males with CD and UC and related controls (control, *n* = 12; CD, *n* = 19; UC, *n* = 24). Representative immunoblots of GPER, ERα and ERβ (**g**). Values are means ± SEM; * *P* < 0.05, ** *P* < 0.01 vs. control.

In men examined in this study, the expression pattern of estrogen receptors substantially resembles that observed in IBD patients without considering sex and age. A statistically significant increase of GPER mRNA expression in men with CD (*P* = 0.020) compared to the control group was observed (Figure 4a). In contrast, in colon samples obtained from men with UC, the absence of GPER mRNA expression changes was shown. Interestingly, a statistically significant up-regulation of GPER protein level in both IBD types, i.e., CD and UC (*P* = 0.006 and *P* = 0.008, respectively) was demonstrated (Figure 4b). Higher, statistically significant expression at both the ERα transcript and protein levels was also demonstrated in colon samples taken from male patients with CD and UC (*P* = 0.005 and *P* = 0.006 for mRNA, respectively; *P* = 0.006 and *P* = 0.010 for protein, respectively) (Figure 4c,d). Despite the absence of statistically significant disturbances of the relative ERβ mRNA and protein level, a downward trend can be observed in male CD patients (Figure 4e,f).

### 2.5. Estrogen Receptors Expression in Women with IBD under the Age of 50

**Figure 5:**
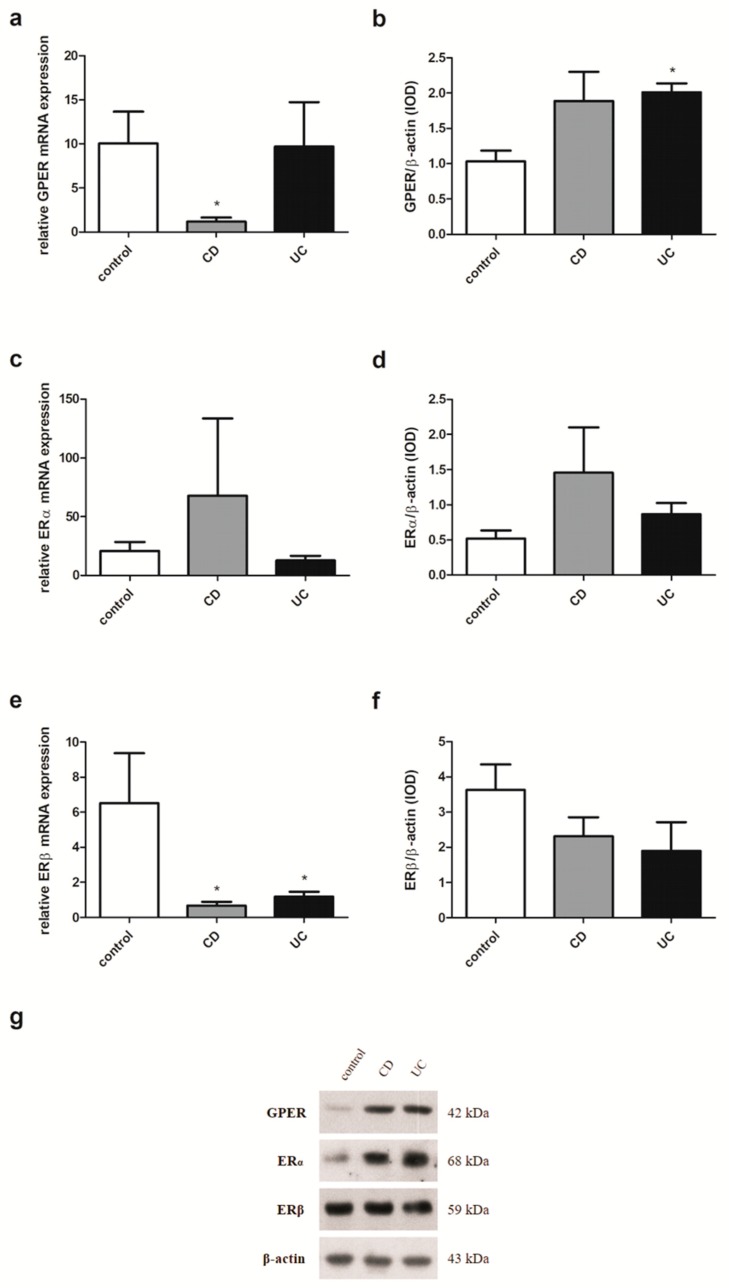
Expression of GPER (**a**,**b**), ERα (**c**,**d**) and ERβ (**e**,**f**) at the mRNA (**a**,**c**,**e**) and protein level (**b**,**d**,**f**) in females with CD and UC under the age of 50 years and related controls (control, *n* = 9; CD, *n* = 7; UC, *n* = 12). Representative immunoblots of GPER, ERα and ERβ (**g**). Values are means ± SEM; * *P* < 0.05 vs. control.

Analysis of the expression of estrogen receptors in the material from women with IBD showed a decrease of GPER mRNA expression in CD (*P* = 0.047) and no changes in UC patients under the age of 50 compared to the control group (Figure 5a). However, women with IBD under the age of 50 were characterized by overexpression of GPER protein although a statistical significance was detected only for UC (*P* = 0.021) (Figure 5b). No statistically significant differences were found in the expression of ERα in women with IBD under the age of 50 (Figure 5c,d). Statistically significant down-regulation of ERβ transcript in both IBD subtypes (*P* = 0.013 for CD and *P* = 0.035 for UC) in women under the age of 50 compared to the control group was shown (Figure 5e). A lower, but not statistically significant, ERβ protein level was also observed in colon samples collected from female patients under 50 years both with CD and UC (Figure 5f).

### 2.6. Estrogen Receptors Expression in Women with IBD over the Age of 50 

**Figure 6:**
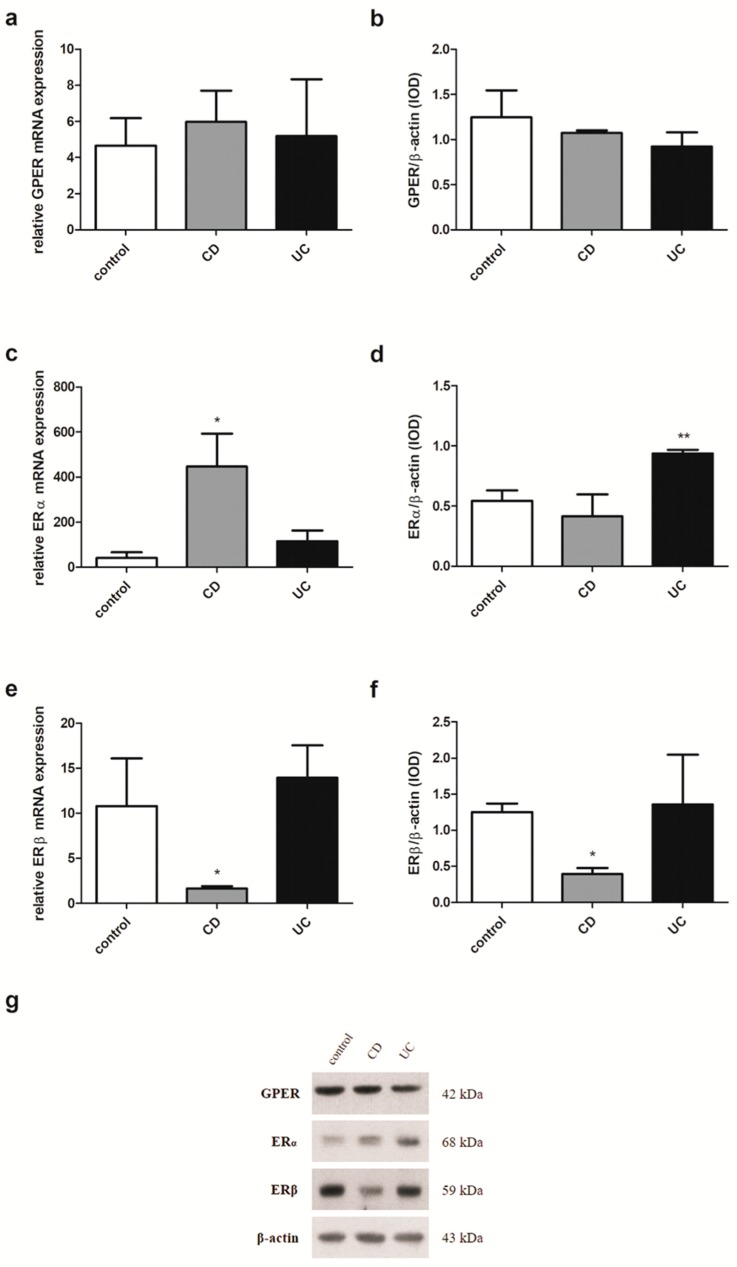
Expression of GPER (**a**,**b**), ERα (**c**,**d**) and ERβ (**e**,**f**) at the mRNA (**a**,**c**,**e**) and protein level (**b**,**d**,**f**) in females with CD and UC over the age of 50 years and related controls (control, *n* = 10; CD, *n* = 5; UC, *n* = 6). Representative immunoblots of GPER, ERα and ERβ (**g**). Values are means ± SEM; * *P* < 0.05, ** *P* < 0.01 vs. control.

In colon samples obtained from women with IBD over the age of 50 when compared to the control group, no statistically significant differences were found in GPER expression (Figure 6a,b). Statistically significant up-regulation of ERα mRNA expression was observed in women with CD over the age of 50 (*P* = 0.020) but not in women with UC in relation to the control group (Figure 6c). However, in the case of protein analysis, women with UC over the age of 50 were characterized by a statistically significant higher protein level of ERα (*P* = 0.003) (Figure 6d). No changes were observed in the ERα protein level in women with CD over the age of 50 compared to the control group. In the case of ERβ analysis, a statistically significant decrease of both mRNA and protein level was identified in samples taken from women with CD over the age of 50 (*P* = 0.027 and *P* = 0.029, respectively) (Figure 6e,f). The relative ERβ mRNA and protein level in UC was at a similar level to that in the control group.

### 2.7. Estrogen Receptor ERα Spliced Variants Expression in IBD Patients

**Figure 7:**
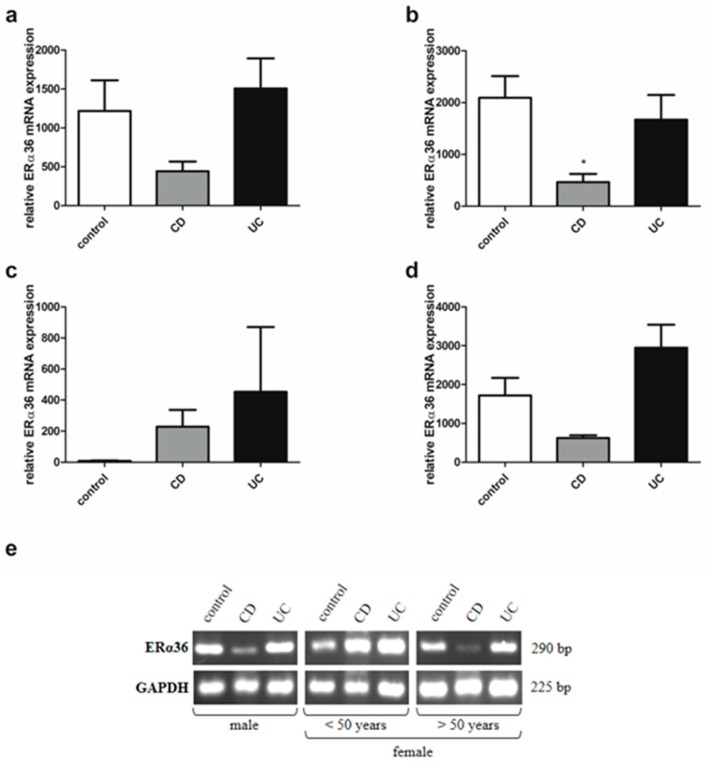
Expression of ERα36 at mRNA level in CD and UC patients and the control group regardless of sex and age (**a**; control, *n* = 31; CD, *n* = 31; UC, *n* = 42), males with CD and UC (**b**; control, *n* = 12; CD, *n* = 19; UC, *n* = 24), females with CD an UC under the age of 50 years (**c**; control, *n* = 9; CD, *n* = 7; UC, *n* = 12), females with CD and UC over the age of 50 years and related controls (**d**; control, *n* = 10; CD, *n* = 5; UC, *n* = 6). Representative agarose gel image of ERα36 expression (**e**). Values are means ± SEM; * *P* < 0.05 vs. control.

**Figure 8:**
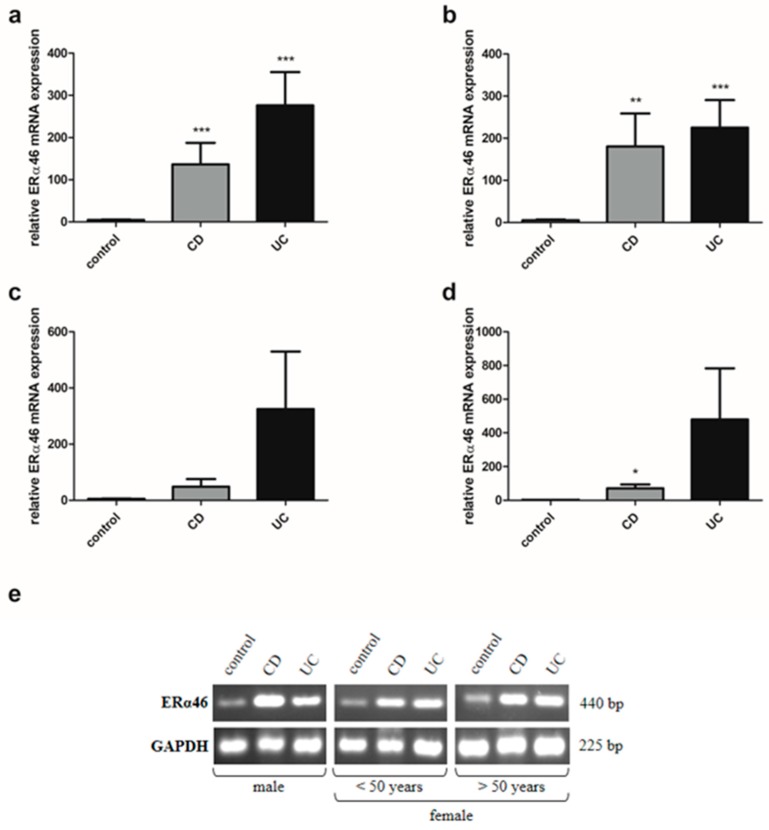
Expression of ERα46 at mRNA level in CD and UC patients and the control group regardless of sex and age (**a**; control, *n* = 31; CD, *n* = 31; UC, *n* = 42), male with CD and UC (**b**; control, *n* = 12; CD, *n* = 19; UC, *n* = 24), females with CD an UC under the age of 50 years (**c**; control, *n* = 9; CD, *n* = 7; UC, *n* = 12), females with CD and UC over the age of 50 years and related controls (**d**; control, *n* = 10; CD, *n* = 5; UC, *n* = 6). Representative agarose gel image of ERα46 expression (**e**). Values are means ± SEM; * *P* < 0.05, ** *P* < 0.01, *** *P* < 0.001 vs. control.

Analysis of estrogen receptors in IBD patients also showed a disruption in the expression of ERα spliced variants, i.e., ERα36 and ERα46 in a sex- and age- dependent manner. There was a statistically significant reduction of ERα36 transcript in colon samples taken from men with CD (*P* = 0.029) in relation to the control group (Figure 7b). Similar to the men with IBD, the ERα36 mRNA expression pattern can be seen in women with IBD over the age of 50 (Figure 7d). In contrast, higher, but not statistically significant, expression of ERα36 mRNA was found in women with CD under the age of 50 compared to the control group (Figure 7c). In the case of ERα46, mRNA expression was shown to increase significantly in men with both types of IBD (*P* = 0.002 and *P* = 0.001 for CD and UC, respectively) compared to the control group (Figure 8b). A similar trend was observed in women with CD under and over the age of 50, but only in women with CD over the age of 50, the increase was statistically significant (*P* = 0.028) (Figure 8c,d).

## 3. Discussion

The pathogenesis of inflammatory bowel diseases remains elusive. There are a few main factors responsible for development of IBD, such as genetic and environmental factors which can lead to epithelial barrier dysfunction and dysregulation of immune response [23,24,25]. On the other hand, there is evidence of the anti-inflammatory effects of estrogens, especially the most active form, i.e., 17β-estradiol [16]. Bábičková et al. [26] found differences related to sex in dextran sodium sulfate (DSS)-induced experimental colitis that suggest a protective role of 17β-estradiol in the development and severity of disease. Male mice were found to be more prone to colitis, which was manifested by worse microscopic and stool score, colon shortening and a higher level of TNF-α in relation to DSS-treated females. We identified that the serum level of 17β-estradiol in both male and female patients with IBD under and over the age of 50 remains within the normal range. Similarly, our analysis revealed no changes in CYP1A1 protein level in CD and UC regardless of the sex and age of the patients. Next, the same level of SULT1E1 in men and women with IBD irrespective of age was observed. CYP1A1 and SULT1E1 are enzymes involved in estrogen catabolism. CYP1A1 is responsible for the hydroxylation of estrogens to 2-hydroxyestrogens which bind to estrogen receptors with less affinity compared to 17β-estradiol. SULT1E1 takes part in the sulfation pathway where it is responsible for the conversion of estrogens to inactive metabolite by conjugation of a sulfo-group to estrone and 17β-estradiol [27].

To elucidate the involvement of estrogen signaling mediated *via* estrogen receptors in IBD, we investigated the local intestine expression pattern of G protein-coupled estrogen receptor (GPER), and nuclear estrogen receptors, ERα and ERβ, as well as spliced variants of wild type ERα – ERα36 and ERα46. Most of the research so far has focused on the role of ERβ in the gastrointestinal tract. It was estimated that ERβ plays an important role in the colon and appears to be responsible for several processes involved in the physiology and pathophysiology of the gastrointestinal tract. Based on immunohistochemical analysis reduced ERβ protein level in intestinal mucosa from patients with active CD and UC as compared to those in remission and healthy controls has been demonstrated [19]. Pierdominici et al. [19] also demonstrated lower ERβ expression in peripheral blood T cells in IBD patients with active disease and patients not responding to anti-TNF-α therapy. A higher pro-inflammatory level of cytokines in the plasma of these patients was observed, but only an inverse correlation of IL-6 with ERβ expression was found. In Caco-2 cell-derived epithelium, ERβ down-regulation after IL-6 supplementation was confirmed. It has been shown that the immunomodulatory action of estrogens is partially related to ERβ. However, Armstrong et al. [28] demonstrated inflammation score reduction in the middle and distal colon in 2, 4, 6-trinitrobenzensulfonic acid (TNBS)-induced murine model of acute colon inflammation not only after 17β-estradiol treatment, but also in TNBS-treated mice with the absence of ERβ. Additionally, 17β-estradiol supplementation caused down-regulation of pro-inflammatory cytokines in both models, but in ERβ *knock-out* mice only a statistically significant reduction of IL-6 and INF-γ was found. It has also been demonstrated that enriched nutritional formulation with ERβ agonist and anti-inflammatory properties may prevent inflammation-associated colorectal cancer in an animal model [29,30].

Although Pierdominici et al. [19] showed that in T cells from IBD patients, a reduction in ERβ expression was accompanied by a significant increase in ERα expression, no differences were observed in the ERα level between patients with CD an UC. Looijer-van Langen et al. [20] also observed no differences between males and females in the level of ERβ mRNA in the colonic biopsies from IBD patients. However, it has been suggested that the serum balance of nuclear estrogen receptors may be crucial for intestine homeostasis and a lower value of ERβ/ERα ratio may be useful for the monitoring of CD but not UC activity. Down-regulation of the ERβ/ERα ratio in serum was demonstrated in CD patients with active disease in relation to CD patients in remission [31]. The reduction of ERβ expression and the concomitant increase of ERα in peripheral blood T cells in patients with IBD, as well as the ERβ/ERα ratio in the serum of IBD patients, have been shown to be not related to the gender and age of the patients [19,31]. However, in our investigations involving more than twice as many patients as in the mentioned studies, we observed a deregulation of the expression of all studied estrogen receptors in the colonic mucosa of both CD and UC patients in a gender and age-dependent manner. Up-regulation of GPER and ERα has been documented in intestinal samples obtained from men with CD and UC. In the case of women, the dysregulation of estrogen receptor expression appears to depend not only on the gender but also on the age of patients. Women under the age of 50 were characterized by lower mRNA expression of GPER in CD. However, at the protein level, GPER up-regulation was observed in UC. There is well-known discrepancy between the level of mRNA and the predicted level of the encoded protein in a particular cell. This was confirmed by a study of global transcriptomics and proteomic analysis, which showed that only approximately 30% of the changes in mRNA levels could be correlated with protein levels [32]. Undoubtedly, post-transcriptional modifications have an impact on the activity and stability of the proteins and adds more complexity to the protein. There were no significant differences in the ERβ protein level between women with CD or UC under the age of 50 and the control group, although a statistically significant reduction in the expression of this receptor at the transcript level was found. However, in women with CD over 50 years of age, a statistically significant decrease of ERβ was stated at both the mRNA and protein levels. A higher statistically significant ERα protein level has been documented in women with UC over the age of 50, while no alteration in GPER expression was observed in women with IBD over the age of 50 years.

There is no doubt that not only ERβ but also ERα and GPER are important for the proper architecture and functioning of colon crypts [33]. ERβ is thought to negatively regulate the activity of ERα [34,35,36]. Kang et al. [37] indicated that down-regulation of ERα36 at the transcription level is mediated *via* GPER. In our previous study we showed that GPER is overexpressed in colon samples obtained from male mice treated with TNBS. However, administration of 17β-estradiol or G-1, a selective GPER agonist, reduced GPER expression at the mRNA and protein level and was associated with improved colitis scores as well as CRP protein level. In addition, the treatment of CD mice with GPER agonists or antagonists was associated with changes in the level of expression in the colon of nuclear estrogen receptors, i.e., ERα and ERβ [22]. Several reports indicate that GPER may play a significant role in immune response [38,39]. Differences in GPER protein levels between non-inflamed and inflamed area in mucosa samples of CD were demonstrated [40]. Additionally, GPER was found to be well distributed in immune cells such as macrophages, neutrophils, B and T cells [41]. Interestingly, a GPER-mediated decrease of phagocytic activity and nitric oxide production during LPS-induced microglial activation in N9 cells was observed [42]. Next, Zhao et al. [43] have shown that G-1, a selective agonist of GPER is able to reduce pro-inflammatory cytokines such as interleukin-1β (IL-1β) and TNF-α in the primary microglia culture.

The results collectively indicate that in CD and UC, alterations of estrogen receptor expression may be associated with the sex and age of patients. Our study shows the complexity of estrogen signaling in the pathophysiology of IBD and gives an insight into the potential mechanisms by which gender and age-related differences in intestinal inflammation can arise. Understanding the mechanisms related to the participation of estrogen receptors in inflammatory bowel diseases depending on the gender and age of the patient may be important for improving the treatment of CD and UC. Furthermore, IBD represents a risk factor for the development of colorectal cancer, and inflammation-associated estrogen receptor dysregulation might be one of the factors linking chronic intestinal diseases to neoplastic transformation.

## 4. Materials and Methods

### 4.1. Study Group and Colon Mucosa Sample Collection

In total, 73 patients with IBD (CD, *n* = 31; UC *n* = 42) and 31 sex and age-related controls were enrolled in the study. The demographic characteristics of patients are shown in Table 3. IBD was evaluated based on clinical, radiological, endoscopical and histological criteria recommended by the European Crohn’s and Colitis Organization. IBD samples were obtained from non-inflamed regions of the colon from patients before the initiation of therapy. Control biopsy specimens were obtained from routine colonoscopy screenings of IBD-free patients. Women were not OC or HRT users. Material was collected by gastroenterologists from the Department of Digestive Tract Disease, Faculty of Medicine at the Medical University of Lodz, Poland. Serum and forceps endoscopic biopsies of colon were taken during hospitalization. The material was frozen and kept at −80 °C for further analysis. The study was conducted in accordance with the ethical principles of the 1975 Declaration of Helsinki and the independent Bioethics Committees of the Medical University of Lodz and the University of Lodz approved the study protocols. All participating subjects gave written, informed consent prior to enrollment.

### 4.2. Enzyme-Linked Immunosorbent Assay

For determination of 17β-estradiol levels in human serum, a dedicated commercially available kit (Demeditec Diagnostics, Kiel, Germany) was used. Ninety-six-well plates were coated with 25 μL of calibrators (0, 25, 75, 225, 675, 2000 pg/mL), low positive control (95 pg/mL), high positive control (252 pg/mL) and samples and incubated over 60 min at room temperature on a plate shaker (>600 rpm). 100 μL of enzyme conjugate was added and the plates were incubated as above. Thereafter, the plates were washed four times with wash solution and 200 μL of substrate solution was added to each well. After 30 min, the reaction was stopped by adding 50 μL of stop solution.

For determination of CYP1A1and SULT1E1 level in human serum ninety-six-well plates were coated overnight at 4 °C with 100 μL of antigen in 50 mM carbonate buffer, pH 9.6. Subsequently, the plates were incubated with commercially available primary antibodies (dilution 1:100, Santa Cruz Biotechnology, Dallas, TX, USA) against CYP1A1 (sc-393979) and SULT1E1 (sc-376009) for three hours at room temperature. Next, the plates were incubated for one hour at room temperature with secondary antibodies (dilution 1:1000) coupled with horseradish peroxidase (HRP; Thermo Scientific, Waltham, MA, USA). *O*-phenylenediamine dihydrochloride (OPD; Sigma Aldrich, Munich, Germany) was used as a substrate for HRP. The reaction with OPD was stopped with 40% H_2_SO_4_. The optimal concentration for each examined protein and antibody was evaluated by titration. The same protocol was used for negative controls (without antigen, primary or secondary antibody). Absorbance was read at 450 nm in a Micro Plate Reader (Bio-Rad, Hercules, CA, USA). All experiments were performed in triplicate.

### 4.3. RNA Isolation

RNA extraction was performed using commercially available TRIsure^TM^ reagent (Bioline, London, UK). Tissue samples were minced and homogenized in TRIsure^TM^. After centrifugation and phase separation, aqueous phase was mixed 3:1 (v/v) with isopropanol and loaded into the column. Subsequent steps were conducted according to manufacturer’s protocol (microRNA Concentrator; A&A Biotechnology, Gdynia, Poland). The purity and quantity of RNA was estimated spectrophotometrically with BioPhotometer Plus (Eppendorf, Hamburg, Germany). The RNA was characterized with A_260_ nm/A_280_ nm ratio, which was in the range of 1.70–2.00.

### 4.4. Real-Time PCR

cDNA synthesis was performed with a High-Capacity cDNA Reverse Transcription Kit (Applied Biosystems, Waltham, MA, USA) in accordance with the manufacturer’s protocol. 1 μg of RNA was used in a reverse transcription reaction with the following incubation steps: 25 °C for ten minutes, 37 °C for 120 min and 85 °C for five minutes. Quantification of mRNA expression was performed using the real-time PCR method with FAM dye-labeled TaqMan^®^ probes (Applied Biosystems, Waltham, MA, USA) and specific primers (Sigma Aldrich, Munich, Germany). The reaction mixture consisted of cDNA, TaqMan^®^ Master Mix II, no UNG, TaqMan^®^ Assays (GPER: Hs00173506_m1, ERα: Hs00174860_m1, ERβ: Hs01100358_m1, IL-6: Hs00985639_m1, IL-10: Hs00961622_m1, CCL18: Hs00268113_m1, TNF-α: Hs01113624_g1, NFκB (RELA): Hs00153294_m1, GAPDH: Hs99999905_m1), and RNase-free water in total volume of 10 μL. For ERα36, ERα46 and GAPDH, the mixture consisted of cDNA, PowerUp^TM^ SYBR^TM^ Green Master Mix (Applied Biosystems, Waltham, MA, USA), forward primer, reverse primer and RNase-free water in total volume of 10 μL. Sequences of primer sets were designed using bioinformatic databases such as NCBI gene, ENSEMBL, MUSCLE and Primer-BLAST and are shown in Table 4. Cycle parameters for TaqMan^®^ Assays were as follows: initial denaturation at 95 °C for ten minutes, followed by 40 cycles of sequential incubations at 95 °C for 15 s and at 60 °C for one minute. Cycle parameters for the reaction with the PowerUp^TM^ SYBR^TM^ Green Master Mix were as follows: UDG activation at 50 °C for two minutes and Dual-Lock^TM^ DNA polymerase at 95 °C for two minutes, followed by 40 cycles of sequential incubations at 95 °C for 15 s and at 60 °C for one minute and the obtained results were normalized to the expression of GAPDH. Real-time PCR products were verified during agarose gel electrophoresis. All experiments were performed in triplicate. The reaction was performed using a Mastercycler^®^ep Realplex4s (Eppendorf, Hamburg, Germany). The fluorescent dye emission was a function of the cycle number. The initial amount of the template was evaluated as a Ct parameter. Ct value corresponded to the threshold cycle number at which PCR amplification reached a significant threshold. The relative expression level was calculated as 2^−∆Ct^ × 1000.

### 4.5. Western Blot Analysis

Proteins were isolated in 100 μL of the radioimmunoprecipitation assay buffer (RIPA; 50 mM Tris/HCl, pH 7.6; 150 mM NaCl; 1% Triton X-100; 0.1% SDS; 1% sodium deoxycholate; 2 mM EDTA) supplemented with 1mM phenylmethanesulfonyl fluoride (PMSF) using an ultrasonic homogenizer (Sonic&Meterials, Newtown, CT, USA). The homogenates were cleared by centrifugation at 5000 rpm for ten minutes. Total protein concentration was evaluated in each sample in triplicate using the Lowry protocol. 10 μg of protein samples were separated on 8% polyacrylamide gels in electrophoresis buffer (25 mM Tris; 192 mM glycine; 0.1% SDS) and electrotransferred onto polyvinyl difluoride (PVDF) membranes (pore size 0.45 μm, Thermo Scientific, Waltham, MA, USA) in transfer buffer (25 mM Tris; 192 mM glycine; 20% methanol) using the wet system. Membranes were blocked 60 min in 5% casein and incubated overnight at 4 °C with commercially available (Abcam, Cambridge, UK) primary antibodies (dilution 1:1000) against GPER (ab39742), ERα (ab75635) or ERβ (ab3576). Subsequently, the membranes were incubated with secondary antibodies (dilution 1:5000) coupled with HRP (31460, Thermo Scientific, Waltham, MA, USA). The optimal concentration of antibodies was selected before the final experiment. After stripping with a buffer containing 10% SDS; 0.5 M Tris/HCl, pH 6.8; 0.8% β-mercaptoethanol, a separate analysis for each sample was performed using β-actin antibodies conjugated with horseradish peroxidase (HRP) (dilution 1:1000, sc-47778, Santa Cruz Biotechnology, Dallas, TX, USA). Immunereaction was visualized using Clarity^TM^ Western ELC Substrate (Bio-Rad, Hercules, CA, USA) and X-ray films (Fujifilm, Tokyo, Japan). The intensities of the visualized signals were analyzed densitometrcially by using Gel Pro Analyzer v3.0 for Windows (Media Cybernetics, Rockville, MD, USA).

### 4.6. Statistical Analysis

Statistical analysis was performed using GraphPad Prism 5.0 (GraphPad Software Inc., San Diego, CA, USA). All data are presented as means ± standard error of mean (SEM). The non-parametric Mann-Whitney U test was used for comparison of the studied groups. *P*-values < 0.05 were considered statistically significant.

## Figures and Tables

**Table 1:** Laboratory findings of control subjects and IBD patients enrolled in the study.

Parameters (unit)	Control	CD	UC
White blood cell (G/L)	8.6 ± 0.2	9.1 ± 1.1	8.4 ± 0.8
Neutrophil (G/L)	5.9 ± 0.2	6.9 ± 1.1	5.4 ± 0.6
Lymphocyte (G/L)	1.8 ± 0.3	1.4 ± 0.1	1.8 ± 0.2
Monocyte (G/L)	0.8 ± 0.6	1.0 ± 0.4	0.7 ± 0.1
Eosinophil (G/L)	0.18 ± 0.01	0.14 ± 0.02	0.14 ± 0.03
Basophil (G/L)	0.05 ± 0.01	0.03 ± 0.01	0.05 ± 0.02
Red blood cell (T/L)	4.4 ± 0.3	4.3 ± 0.1	4.3 ± 0.1
Hemoglobin (g/dL)	13.9 ± 0.4	12.7 ± 0.3	12.7 ± 0.3
Sodium (mmol/dL)	139.6 ± 4.0	137.6 ± 0.5	137.0 ± 1.5
Potassium (mmol/dL)	4.3 ± 0.2	4.0 ± 0.1	4.0 ± 0.1
C-reactive protein (CRP; mg/dL)	1.1 ± 0.2	31.8 ± 9.1 **	35.4 ± 11.3 ***

Values presented as a means ± SEM; ** *P* <0.01, *** *P* <0.001 vs. control.

**Table 2:** Relative mRNA expression level of selected biomarkers of inflammation in control subjects and IBD patients enrolled in the study.

Inflammation Biomarkers	Control	CD	UC
Interleukin-6 (IL-6)	4.11 ± 1.25	30.98 ± 13.18 **	50.33 ± 21.14 *
Interleukin-10 (IL-10)	1.57 ± 0.53	2.21 ± 0.78	6.00 ± 2.83
C-C chemokine motif ligand 18 (CCL18)	1.08 ± 0.35	4.94 ± 1.64	2.16 ± 0.36 *
Tumor necrosis factor-α (TNF-α)	4.64 ± 1.01	5.62 ± 1.40	5.89 ± 1.76
Nuclear factor kappa-light-chain-enhancer of activated B cells (NFκB)	102.10 ± 11.21	138.10 ± 34.29	116.90 ± 29.76

Values presented as a means ± SEM; * *P* <0.05, ** *P* <0.01 vs. control.

**Table 3:** Demographic profiles of control subjects and IBD patients enrolled in the study.

	Subjects and Age of Subjects	Control	CD	UC
Total	*n*	31	31	42
	mean ± SD	63.08 ± 15.79	48.40 ± 17.55	45.22 ± 12.63
	range	22–86	29–86	22–81
Male	*n*	12	19	24
	mean ± SD	63.27 ± 12.67	49.43 ± 48.90	43.24 ± 11.07
	range	32–83	29–86	29–65
Female under the age of 50 years	*n*	9	7	12
	mean ± SD	41.60 ± 9.52	36.14 ± 7.92	39.43 ± 6.26
	range	22–49	29–48	22–47
Female over the age of 50 years	*n*	10	5	6
	mean ± SD	73.60 ± 9.69	66.25 ± 3.30	65.00 ± 9.59
	range	57–86	64–71	52–81

**Table 4:** Primer sequences and the length of the amplicons of ERα splice variants and the housekeeping gene.

Gene	Primer	Sequence	Amplicon Length (bp)
ERα36	Forward Reverse	CAAGTGGTTTCCTCGTGTCTAAAG TGTTGAGTGTTGGTTGCCAGG	290
ERα46	Forward Reverse	GTGCTCCCCAAATTTCCTTTCA GCCTTCGCCATTGAAGTCAC	440
GAPDH	Forward Reverse	CTTCGCTCTCTGCTCCTCCTGTTCG ACCAGGCGCCCAATACGACCAAAT	225
